# Peritoneal histopathological changes and cultures after autogenous fecal peritonitis induced in elderly rat model: response to intravenous use of meropenem and intra-abdominal inoculation of 10% aqueous extract of *Schinus Terebinthifolius Raddi* (Anacardiaceae)

**DOI:** 10.1590/acb400125

**Published:** 2024-12-20

**Authors:** Carlos Alberto Figueiredo, Celia Maria Machado Barbosa Castro, Guilherme Veras Mascena, Gustavo Ithamar Souto Maior, Tharcia Kiara Beserra Oliveira, Valéria Wanderley Pinto Brandão Marquis, Carlos Teixeira Brandt

**Affiliations:** 1 Universidade Federal de Pernambuco – Medicina Tropical – Recife (PB) – Brazil.; 2 Universidade Federal de Pernambuco – Hospital das Clínicas – Recife (PB) – Brazil.; 3 Centro Universitário Facisa – Campina Grande (PB) – Brazil.

**Keywords:** Peritonitis, Aged, Carbapenems, Rats, Histology, Anacardiaceae

## Abstract

**Purpose:**

To evaluate the peritoneal histopathological changes and culture after the use of intravenous meropenem and intra-abdominal inoculation of 10% aqueous extract of anacardiaceae, in elderly rat model after autogenous fecal peritonitis induced.

**Methods:**

Thirty 18-month-old Wistar rats received induction of autogenous fecal peritonitis and then were stratified into two groups: study I, treated with meropenem (40 mg/kg); and study II, treated with meropenem (40 mg/kg) and intraperitoneal 10% aqueous extract of anacardiaceae. Animals were monitored for 15 days until euthanasia. Peritoneal fragments were collected for histopathological and culture. The study was approved by Ethics Committee.

**Results:**

None study-II animals died, while in study I, one died before euthanasia. In study II, 20% of the animals showed histopathological changes, none positive peritoneal culture, but one blood culture was positive (10%). In study I, 50% of the animals presented histopathological changes, 40% positive peritoneal cultures, and 50% positive blood cultures. All results when evaluated in the morbidity score showed better outcome for study-II group (*p* = 0,175).

**Conclusion:**

The use of meropenem associated with intraperitoneal 10% aqueous anacardiaceae extract after induction of autogenous fecal peritonitis in elderly rats showed better outcome in the set of histopathological changes, negative peritoneal and blood cultures, when compared with the use of meropenem isolated.

## Introduction

Intraperitoneal infections affecting persons aged ≥ 65 years old are difficult to diagnose etiologically, unusual presentation, increased severity and with a more reserved prognosis than in young individuals, leading to systemic septic conditions^1^.

The management of antibiotic therapy for peritonitis has faced challenges with the emergence of multi-resistant bacteria, often requiring combined use of medications, as well as intra-abdominal interventions, to remove the focus of infection^2^^,^ 3.

The peritoneum serves as a natural barrier, like a thin membrane, which internally covers the entire anterior abdominal wall and the abdominal viscera. Peritonitis, due to the inflammatory process, alters the permeability of the peritoneal membrane by damaging both the superficial mesothelial layer of the peritoneum and the submesothelial connective tissue and vasculature^4^^,^ 5.

There has been a frequent search for agents with bactericidal and anti-inflammatory action for intraperitoneal use during cavitary toilet surgeries, such as: mezalazine, dexamethasone, and extracts of medicinal plants^6^^,^ 7.

In this context, Anacardiaceae extract is already widely used as an anti-inflammatory and antibacterial agent in vulvovaginitis, gastritis, and dermatitis, as it has components such as: squinol, masticadienoic acid, biflavonoids, hydroxymicadienoic acid, terebintifolic acid, and ursolic acid^8^^,^ 9.

The objective of the present study was to investigate whether the intrabdominal use of *Anacardiaceae* extract in the surgical treatment of peritonitis, together with broad-spectrum intravenous antibiotic therapy, could act as an anti-inflammatory agent, potentially reducing peritoneal histopathological changes and bactericidal by decreasing the concentration of intra-abdominal bacteria.

## Methods

Thirty male Wistar rats (*Rattus Norvegicus, Rodentia, Mammalia*) at 18 months old, without any disease or injury from the breeding colony of the Faculty Medical Sciences of Campina Grande of the Centro Universitário Facisa, were included.

Initially, peritonitis was induced by intraperitoneal inoculation of a 10% autogenous fecal suspension prepared with 2 g of feces dissolved in 20 mL of 0.9% saline solution, homogenized and filtered with a gauze to remove the large particles.

**Figure 1:**
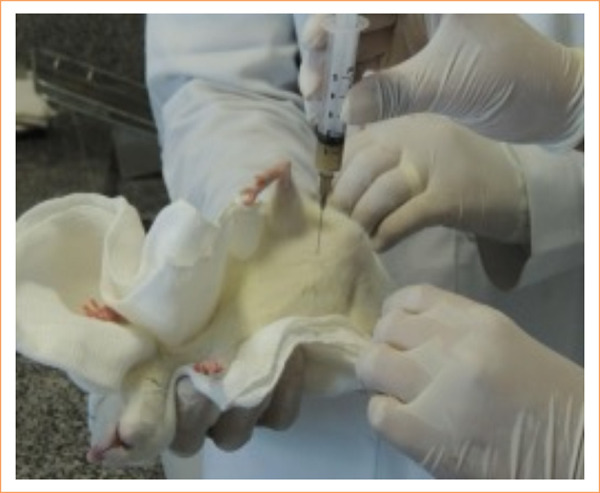
Induction of peritonitis with autogenous fecal solution.

Source: Elaborated by the authors.

The produced solution was injected into the intraperitoneal cavity at the dose of 4 mL/kg of 10% solution of feces (Fig. 1).

The animals were divided into two groups of 10 male rats each: study I and study II. All rats were submitted to intravenous administration of meropenem at the dose of 40 mg/kg six hours after the peritonitis induce. Only the group study II received an intraperitoneal inoculation of 10% aqueous extract of *Anacardiaceae*.

The animals that survived until the 15th day after induction of peritonitis were euthanized with intravenous administration of ketamine hydrochloride at the dose of 50 mg/kg and xylazine at the dose of 10 mg/kg.

The animals that died before the expected follow-up were also submitted to the same evaluated procedures.

**Figure 2:**
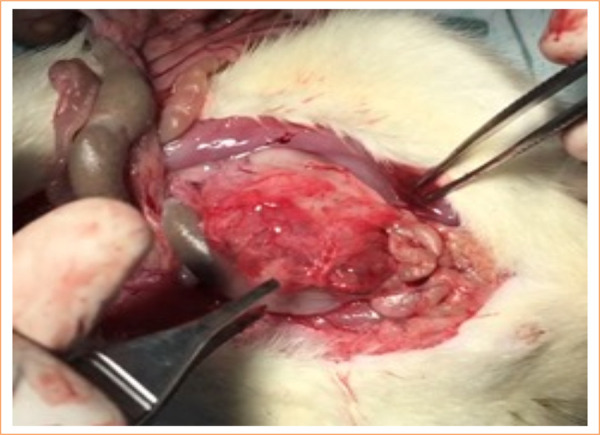
Laparotomy to access the peritoneum.

Source: Elaborated by the authors.

**Figure 3:**
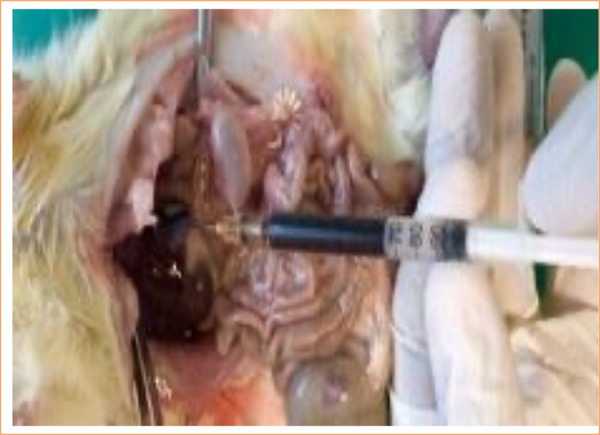
Abdominal aorta puncture to collect blood for culture.

Source: Elaborated by the authors.

Upon accessing the abdominal cavity (Fig. 2), the abdominal aorta was punctured with a 30 × 1.5-mm needle, and 2 mL of blood was aspirated and sent to culture (Fig. 3).

Afterwards, we exercised fragments of the parietal peritoneum for culture and histopathological evaluation. The samples for histopathological evaluation were fixed in 10% neutral formalin solution, placed in paraffin, and stained with hematoxylin and eosin (H&E).

For culture, the peritoneum fragments were inserted into brain heart infusion broth, incubated for six hours in an oven at 37°C, and plated on blood agar and salt mannitol agar. The grown gram-positive bacteria were identified through catalase, coagulase, optochin, and bacitracin testing. Gram-negative bacteria were identified using specific biochemical tests.

To stratify the morbidity of animals in the face of the infectious insult of fecal autogenous peritonitis, a score was used that included the results of blood culture, culture of peritoneal fragments, and histopathological changes, like acute infection (polymorphonuclear), chronic infection (monomorphonuclear), necrosis, and angiogenesis.

### Scores 0–3: death before day 15

0: death from septic shock in the first 24 hours;

1: death between 24 and 48 hours;

2: death between 48 hours and eight days;

3: death between 8 and 15 days.

### Scores 4–9: survival until day 15, followed by euthanasia

4: positive blood culture. Positive peritoneal culture and peritoneal histopathological changes found;

5: positive blood culture. Positive peritoneal culture and peritoneal histopathological changes not found;

6: positive blood culture. Negative peritoneal culture, peritoneal histopathological changes found;

7: negative blood culture. Positive peritoneal culture, peritoneal histopathological changes found;

8: negative blood culture. Negative peritoneal culture, peritoneal histopathological changes found;

9: negative blood culture. Negative peritoneal culture, peritoneal histopathological changes not found.

The samples were of convenience. Quantitative variables were expressed as means and standard deviations. Qualitative variables were expressed in frequencies. Shapiro-Wilk test and Kolmogorov-Smirnov test were used to verify data normality. The Mann-Whitney test was used to assess possible differences between morbidity scores in the two groups. Values of *p* < 0.05 were used to reject the null hypothesis.

## Results

The blood cultures and peritoneal cultures findings are shown in Tables 1 and 2. These results were included in morbidity score for both groups.

**Table 1:** Results of hemocultures

Study I		Study II	
Rat 1	Positive * *Streptococcus viridans* * *Escherichia coli*	Rat 1	Negative
Rat 2	Positive * *Escherichia coli* * *Staphylococcus aureus*	Rat 2	Positive * *Escherichia coli*
Rat 3	Positive * *Escherichia coli*	Rat 3	Negative
Rat 4	Negative	Rat 4	Negative
Rat 5	Negative	Rat 5	Negative
Rat 6	Positive * *Escherichia coli*	Rat 6	Negative
Rat 7	Negative	Rat 7	Negative
Rat 8	Positive * *Escherichia coli* * *Klebsiella pneumoniae*	Rat 8	Negative
Rat 9	Negative	Rat 9	Negative
Rat 10	Negative	Rat 10	Negative

* Bacteria isolated in blood culture. Source: Elaborated by the authors.

**Table 2:** Results of peritoneal cultures

Study I		Study II	
Rat 1	Positive *Escherichia coli	Rat 1	Negative
Rat 2	Negative	Rat 2	Negative
Rat 3	Positive *Escherichia coli	Rat 3	Negative
Rat 4	Negative	Rat 4	Negative
Rat 5	Negative	Rat 5	Negative
Rat 6	Positive *Escherichia coli	Rat 6	Negative
Rat 7	Negative	Rat 7	Negative
Rat 8	Positive *Escherichia coli *Klebsiella pneumoniae	Rat 8	Negative
Rat 9	Negative	Rat 9	Negative
Rat 10	Negative	Rat 10	Negative

* Bacteria isolated in blood culture. Source: Elaborated by the authors.

**Figure 4:**
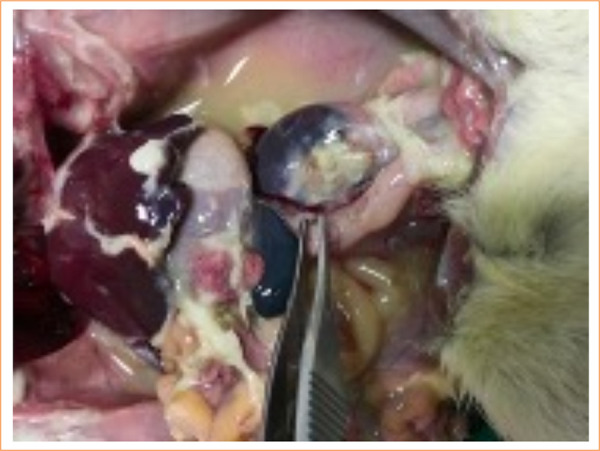
Peritonitis in rat 2 from study-I group that died during follow-up.

Source: Elaborated by the authors.

None of the study-II rats died during follow-up, but in study-I group one animal did (Rat 2) (Fig. 4). This result was included in morbidity score for both groups.

**Figure 5:**
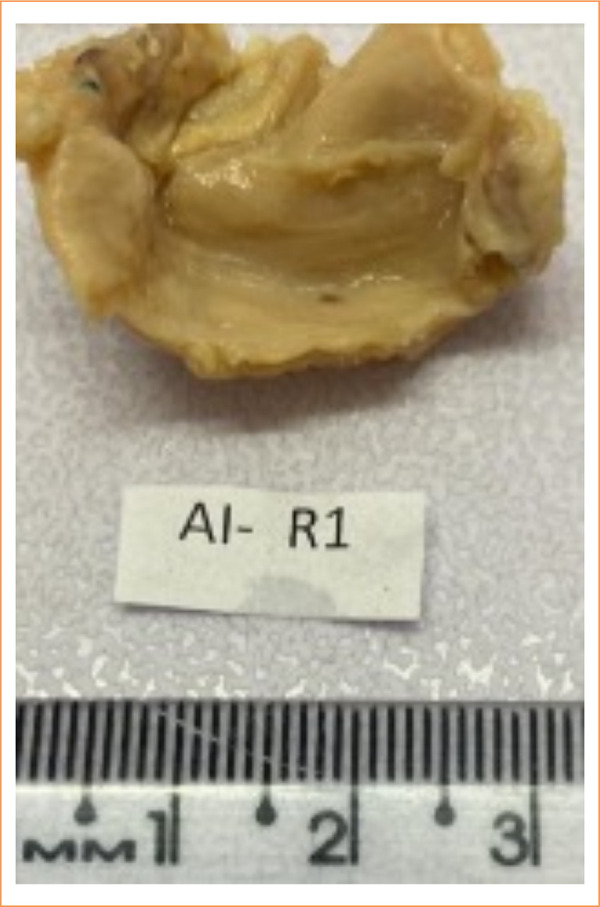
Fragment of peritoneum fixed in 10% neutral formalin solution.

Source: Elaborated by the authors.

**Figure 6:**
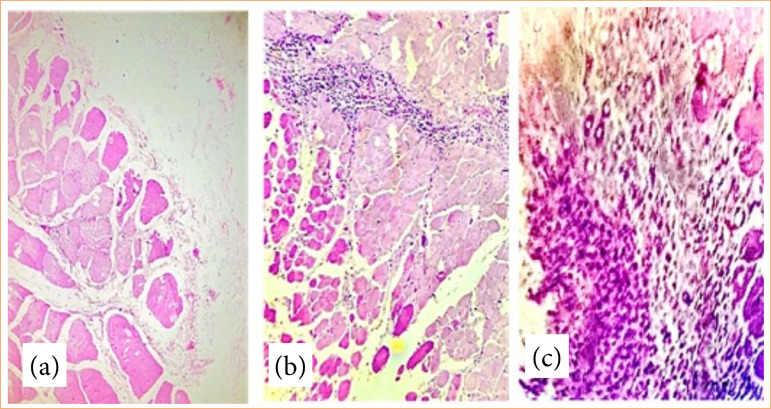
**(a)** Study II: muscle tissue and connective tissue without inflammatory changes; **(b)** study I: perivascular lymphoplasmocytic inflammatory infiltrate dissecting the muscle fibers; **(c)** study II: granulation tissue with angiogenesis, fibroblast proliferation and lymphohistiocytic inflammatory infiltrate.

Source: Elaborated by the authors.

When evaluating the peritoneal histopathological findings, we observed the following alterations: acute infection (polymorphonuclear), chronic infection (monomorphonuclear), necrosis, and angiogenesis. These results were included in morbidity score for both groups (Table 3 and Figs. 5 and 6).

**Table 3:** Results of peritoneal histopathological findings: acute infection (polymorphonuclear), chronic infection (mono morphonuclear), necrosis and angiogenesis.

Study I		Study II	
Rat 1	Positive *Polymorphonuclear *Angiogenesis	Rat 1	Negative
Rat 2	Negative	Rat 2	Positive
Rat 3	Positive *Polymorphonuclear *Angiogenesis *Necrosis *Monomorphonuclear	Rat 3	Negative
Rat 4	Negative	Rat 4	Positive
Rat 5	Negative	Rat 5	Negative
Rat 6	Positive *Polymorphonuclear *Angiogenesis	Rat 6	Negative
Rat 7	Negative	Rat 7	Negative
Rat 8	Positive *Polymorphonuclear *Angiogenesis	Rat 8	Negative
Rat 9	Negative	Rat 9	Negative
Rat 10	Negative	Rat 10	Negative

* Peritoneal histopathological findings. Source: Elaborated by the authors.

**Figure 7:**
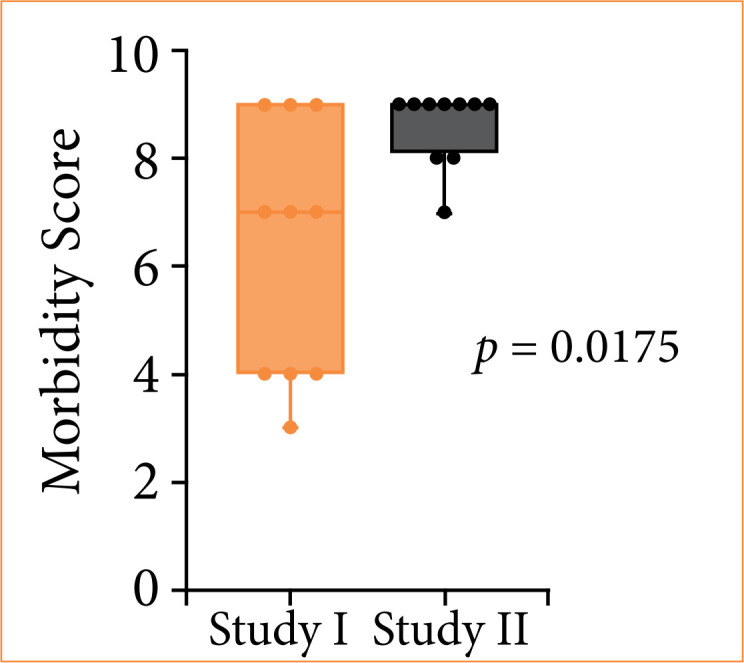
Comparison of morbidity scores in both groups.

Source: Elaborated by the authors.

The mean of the morbidity score of the study I and study II was, respectively: 6.3 ± 0.5 and 8.6 ± 0.4, resulted in a better outcome for the study-II group, with a significant difference between the means (*p* = 0.175) (Table 4 and Fig. 7).

**Table 4:** Results of the means of the morbidity scores in each group.

	Mean Score	*p* < 0.05
Study I	6.3 ± 0.5	*p = 0.0175
Study II	8.6 ± 0.4	

* Mann-Whitney test. Source: Elaborated by the authors.

## Discussion

Most of the experimental models involving peritonitis use the inoculation of intra-abdominal fecal solution in order to mimic intra-abdominal infection site in the human population. Although more than half of the cases of peritonitis occur in the elderly population, few studies use old animals in their methodologies^10^^–^ 12.

In the present study, the production of an experimental model with elderly Wistar rats was satisfactory, based on intraperitoneal inoculation of 10% autogenous fecal solution at the dose of 4 mL/kg, capable of developing an insult of peritonitis without necessarily culminating in death.

In this study, animals that received intravenous infusion of meropenem combined with intraperitoneal inoculation of *Anacardiaceae* extract showed fewer histopathological changes in the peritoneum, suggesting that the peritoneal barrier was kept intact, without increased permeability^13^.

The results of peritoneal fragment cultures with lower positivity in this study suggest that the intraperitoneal use of a *Anacardiaceae* extract for the local treatment of peritonitis as a bactericidal agent is promising, possibly due to the action of terebintifolic acid, a phenol which has significant antimicrobial activity against several strains of bacteria^14^.

The synthesis of silver nanoparticles (AgNPs) derived from medicinal plants represents a promising alternative in the development of antimicrobial agents. In a recent innovative study, the aqueous extract of *Anacardiaceae* leaves proved to be effective in the synthesis of AgNPs, presenting greater antimicrobial potential when compared to the original extract^15^.

Therefore, studies involving sepsis and peritonitis, especially in the elderly, must insist on the challenge of studying new therapeutic approaches, including the evaluation of medicinal plant extracts as an adjuvant in the intraperitoneal removal of the infectious focus.

## Conclusion

The use of meropenem associated with 10% aqueous extract of *Anacardiaceae* intraperitoneally after induction of autogenous fecal peritonitis in elderly rats showed better evolution in morbidity scores that include histopathological and peritoneal changes and negative blood cultures when compared to the use of meropenem alone.

## Data Availability

The data will be available upon request.
